# Impact of climate change on the Himalayan alpine treeline vegetation

**DOI:** 10.1016/j.heliyon.2024.e40797

**Published:** 2024-11-28

**Authors:** Sandeep Kumar, Vinod Prasad Khanduri

**Affiliations:** College of Forestry, Veer Chandra Singh Garhwali Uttarakhand University of Horticulture and Forestry, Ranichauri, 249199, Uttarakhand, India

**Keywords:** Global warming, Krummholz, Timberline, Tree regeneration, Treeline shift

## Abstract

The Himalayan alpine treeline varies depending on altitude and aspects, supporting a variety of plant species. In recent years, climate changes have exerted pressure on the vegetation in this region, challenging its adaptation to rapidly changing environmental conditions. This systematic review commenced by formulating a research question on the impact of climate change on Himalayan alpine treeline vegetation and conducted a thorough literature search, adhering to the PRISMA protocol. The rising temperatures, altered precipitation patterns, and other climate-related factors have initiated an upward shift in the treeline that threatens the unique biodiversity of the region. Indeed, in various parts of the Himalayas, there is evidence of the treeline moving upwards, altering plant regeneration and growing season, and impacting soil properties. There is a shift of vegetation ranging from 0.80 to 503.00 m in Himalayan treeline regions have been reported in various studies. *Abies spectabilis* and *Betula utilis* are the most sensitive, showing the highest upward shifts due to climate change. The repercussions of climate change on the Himalayan alpine treeline are anticipated to have significant ecological implications. Most species at the Himalayan alpine treeline exhibit poor regeneration status, while some others reveals good, fair, or no regeneration. Consequently, new regeneration patterns are emerging. Changes in soil temperature and physicochemical properties due to climate warming are ultimately affecting Himalayan alpine treeline vegetation. Additionally, shifts in the growing season and phenophases of various tree species have also been observed. The profound and far-reaching impacts of climate change on the Himalayan alpine treeline necessitates implementing mitigation and adaptation strategies to safeguard the delicate alpine ecosystems of the region.

## Highlights

• Emphasizes the critical need to understand how climate change impacts vegetation in the Himalayan alpine treeline region.
• The Himalayan treeline is undergoing remarkable shifts, with key species such as *Abies spectabilis*, *Betula utilis*, and *Rhododendron campanulatum*.
• Climate change significantly impacts plant regeneration and alters soil properties in the treeline region, leading to changes in stand structure and species migration patterns.

## Graphical abstract

**Figure:**
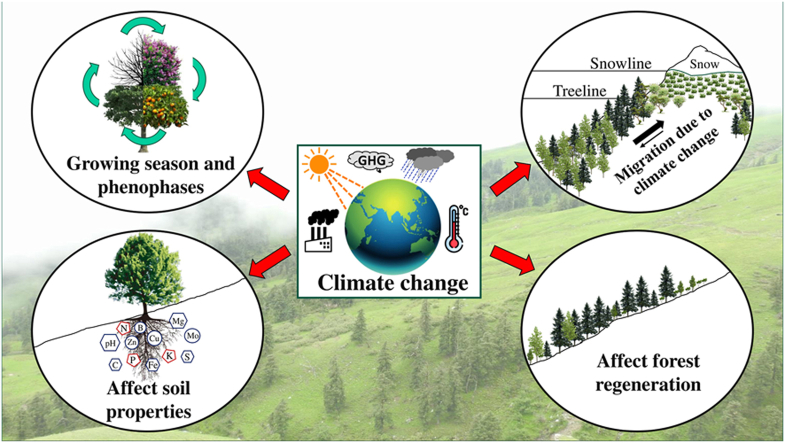

## 1 Introduction:

The Himalayas are one of the most biodiverse regions in the world, supporting tremendous plants, animals and aquatic biodiversity and are considered as home for thousands of plant and animal species [1]. The existence of biodiversity in the young folded mountains is due to the unique combination of factors such as varied topography, complex geological structures, the enormous sizes of the valleys, river gorges, alpine glaciers, varied climatic conditions, and diverse soil types [2]. The impact of climatic variability and global temperature rise is seen all over the world, however, it is particularly high in the Himalayas which is evidenced by the rapid melting of glaciers, loss of snow cover, changes in vegetation cover, loss of biodiversity, irregular weather patterns, and increasing frequency and severity of natural disasters [[3], [4], [5], [6], [7]]. Under the influence of global climate change, the Himalayas are warming at 3 times greater than the global average rate. For example, the temperature of Nepal is rising by 0.6 °C per decade in comparison to that of the rise of the global average of 0.74 °C over the last 100 years [4,8,9], also ascertained the higher number of warm days [10] and affected the surface temperature including lapse rate [11,12]. Shrestha et al. [13] reported that the Himalayas experienced a warming of 1.5 °C between 1982 and 2006, with an average annual increase of 0.06 °C. Specifically, during the season cycles, winter exhibited the highest rise at 0.07 °C per year. Summer showed the lowest increase by 0.03 °C per year. An increase in temperature in the treeline region affects plant phenophases and distribution range, leading to the expansion of treeline species into the adjacent tundra ecosystem [14,15]. According to Joshi et al. [16], Himalayan treelines recorded a higher growing season temperature of 8.4 ± 1.8 °C in the Western Himalaya, 10.3 ± 1.4 °C in Uttarakhand and Central Himalaya, and 7.5 ± 2.7 °C in the Eastern Himalaya. These findings suggest that treelines in the region are experiencing warmer trends, surpassing the global growing season temperature of 6.5 ± 0.8 °C [17].

Climate change refers to the long-term shifts in climatic conditions, including changes in average temperature, precipitation, and other factors like pressure and humidity, usually observed over decades or longer [18,19]. Irregular weather patterns, retreating ice sheets, and rising sea levels are some of the most significant global and local impacts of climate change [20,21]. This change is driven primarily by natural and human activities like fossil fuel combustion, deforestation, and land use changes [22]. These activities increase greenhouse gases, notably carbon dioxide (CO_2_), which traps heat and raises global temperatures [[22], [23], [24]]. Irregular weather patterns, retreating ice sheets, and rising sea levels are among the most significant global and local impacts of climate change [20]. Atmospheric CO_2_ concentrations have significantly increased from 280 ppm in 1750 to over 400 ppm in 2013, reaching 425.55 ppm in July 2024. This increase has contributed to a rise in the global average surface temperature by +1.50 °C in June 2024 compared to the period of 1880–1920 [25]. These temperature increases cause frequent heat waves, altered precipitation, and rising sea levels, which severely impacting biodiversity and causing declines in species populations [26] and prompting the migration of birds, animals, butterflies, and plant species toward cooler areas to find suitable habitats [27,28]. This migration affects individual genotypes to entire communities [29], disrupts life cycles and phenological events of species [30,31], leads to habitat loss, and alters species distribution. Climate change threatens species' adaptability that requires solutions like reducing greenhouse gas emissions, protecting ecosystems, and preparing for inevitable impacts.

**Fig. 1:**
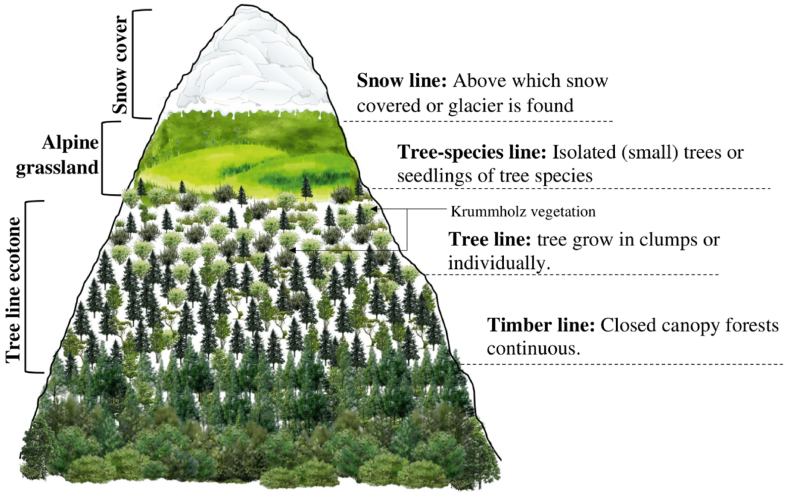
An overview of the Himalayan alpine treeline region, highlighting the key vegetation zones in relation to elevation.

**Fig. 2:**
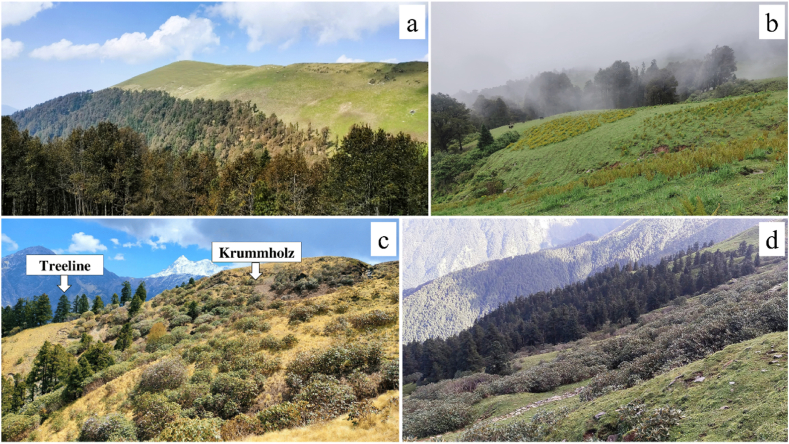
Overview of Himalayan alpine treeline; **a & b**: treeline of Ali-Bedni and Dayara bugyal, Uttarakhand, dominanted by *Quercus semecarpifolia*, elevation 3450 and 3340 m respectively; **c & d**: treeline dominated by *Abies spectabilis* and krummholz of *Rhododendron campanulatum* at Bedani Bugyal (c) and Tungnath (d), Uttarakhand, elevation 3340 and 3350 m respectively.

The alpine treeline is the high-elevational area where closed canopy forests from lower elevations (also called forest lines or timberlines) give way to the open alpine tundra, grasslands and rocky expanses [32]. Korner [33] defines alpine treelines as delineating the low-temperature limit of tree growth, found in mountain ranges globally. Generally, the alpine treeline is located between the tree species line or krummholz (refers to the zone between the treeline and more open alpine vegetation) and the timberline or forest line (upper limit of the closed forest), which roughly corresponds to the areas of dense patches of trees (Fig. 1) [[33], [34], [35]]. In the treeline ecotone, the ‘tree species line’ refers to tree species shorter than 2 m, often multi-stemmed or damaged by extreme weather. These include krummholz, which are dwarfed, crooked trees with deformed shapes [36]. In the Himalayan alpine treeline region, the habit/lifeforms of *Rhododendron campanulatum* is large shrub that reaches heights up to 8 m and forms dense krummholz zones above the treeline. The high-altitude forests in the Himalayas have their own inimitable identity and play a vital role in the functioning of the ecosystem, setting them apart from other types of forests. The Himalayan treeline is a habitat and a vegetative transition zone above which trees cannot grow due to harsh climatic conditions [37,38]. Unlike the latitudinal treeline, this zone occurs at higher elevations and forms one of the highest treelines in the world, stretching over 4000 km across several countries including India, Nepal, Bhutan, China and Pakistan. The treeline in the Himalayas varies depending on location and altitude, ranging from around 3000 to 4000 m [39]. At these elevations, temperatures are generally cool, and precipitation is often limited, leading to a challenging environment for vegetation to thrive. Despite these harsh conditions, this vegetation limit supports a variety of plant species, each adapted to the unique environmental conditions of their location. Moving up the mountain, the treeline transitions into dwarf juniper scrub, characterized by stunted juniper trees taking on a shrub-like appearance. Finally, above this zone, the treeline is composed of low-growing plants such as rhododendrons and dwarf birch that forms the Himalayan alpine krummholz vegetation [36,40], (Fig. 2). The Himalayan treeline represents ecologically sensitive vegetation that plays a decisive role in the regulation of water resources, carbon storage, and biodiversity.

The alpine treelines serve as crucial indicators revealing the swift impact of climate change on forest distribution and tree growth in the Himalayan mountains [41,42]. These phenomena are influenced by factors such as species composition, stand structure, biotic interactions [43], topography and human disturbance [44]. Several treeline dynamics such as extreme climatic circumstances, soil, topography, water stress, frost, snow, wind, limited resources etc. are the most important causes that influence the timberline ecotones [32,37,45]. Climate change, which has become increasingly prominent in the region in recent years, poses a serious threat to the Himalayan treeline ecosystem. Furthermore, changes in precipitation patterns and soil properties significantly impact the distribution of tree species in the treeline ecotone [46,47]. Variations in precipitation, including changes in the amount, frequency, and seasonality of rainfall, can alter soil moisture levels and physicochemical properties, which in turn affect seed germination, seedling survival, and overall tree growth. Research on Himalayan alpine treeline regions often focuses on broad vegetation changes, but the responses of individual tree species to climate change remain underexplored. Short-term field data frequently overlook gradual or sporadic shifts in the treeline, while limited long-term data on vegetation composition, regeneration, and seedling survival hinders accurate predictions. Although general climate effects are recognized broadly, however microclimatic factors such as soil moisture, snowpack and local temperature fluctuations are required to be well studied which significantly influence growth and species composition. Additionally, disturbances like grazing, fire, and human activities along with their combined effects under warming conditions, require further investigations. Information on shifts in phenological events, such as bud formation, leaves flushing, flowering, leaf colouration, leaf fall and biotic interactions to treeline dynamics are scarce. Moreover, research on the socio-economic impacts of treeline shifts on local communities and their climate adaptation strategies is limited. In this article, we aimed to compile all available pieces of information on the Himalayan treeline region, particularly in the context of climate change which can reveal shifts in species composition, treeline position, plant growth patterns, soil properties and regeneration rates over time. Analyzing these factors can shed light on how the treeline has responded to climatic changes over time, which could be helpful for a clearer understanding of the response of climate change to the Himalayan alpine treeline regions and providing a more defined call of action for researchers, environmentalists, local communities, policymakers, and conservationists.

## 2 Methodology and data collection

**Fig. 3:**
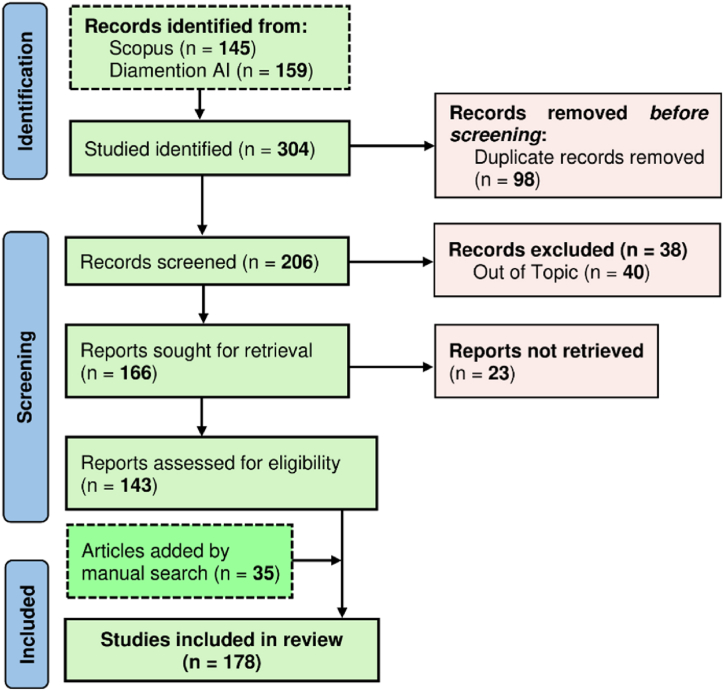
Flow diagram depicting the systematic process of reviewing and selecting articles for inclusion in the study.

**Fig. 4:**
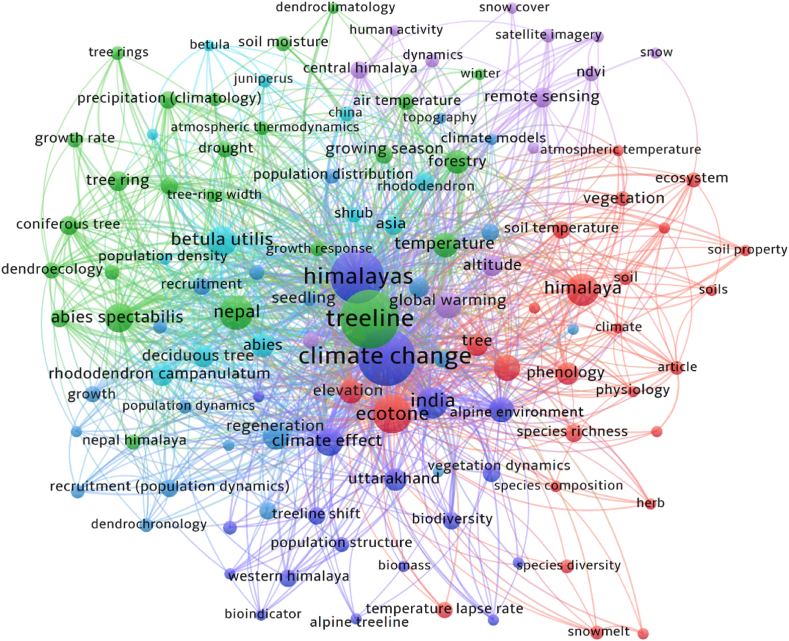
The co-occurrence network of keywords generated through database searches (visualized using VOSviewer).

**Fig. 5:**
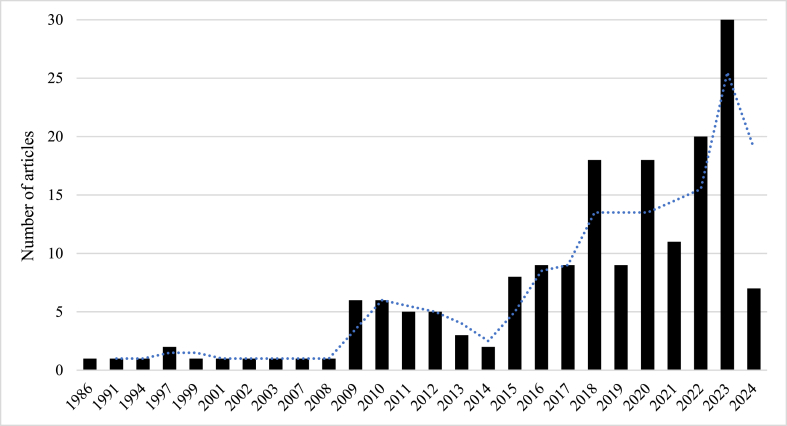
Distribution of the 178 reviewed articles categorized by their publication year.

This systematic review followed a structured approach (1) encompassing the framing of a research question (2) Search and identification of publications (3) screening and assessment of articles (4) summarizations of evidences and finally (5) interpretation and presentation of findings. The research commenced with the question: How does climate change impact Himalayan alpine treeline vegetation? Next, an extensive literature search was conducted using the 'SCOPUS' and 'Dimensions' online databases to ensure the selection of rigorous articles and other published materials. Two distinct sets of search terms, connected by the "AND" and "OR" operators, were used for basic searches. Specifically, the literature search employed the keywords "Treeline" AND "Himalayan" OR "Himalaya" AND "Climate Change" OR "Global Warming" OR "Temperature" OR "Shift" OR "Regeneration". Next, 145 articles from ‘Scopus’ and 159 from ‘Dimensions’ were identified. Publications were screened in the 3rd step to determine if their titles, abstracts and keywords aligned with the review's research questions. Additionally, 35 articles were manually added using several databases i.e. Google Scholar, Web of Science, J–gate, and ResearchGate. Consequently, the PRISMA (Preferred Reporting Items for Systematic Reviews and Meta-Analyses) protocol was selected to ensure the scientific quality of the review [48], understand the review procedures and analyze irrelevant record attrition during the review [49]. Overall, 178 articles were determined to be eligible for the systematic review (Fig. 3, Fig. 5). Following a comprehensive investigation, the collected informations are summarised under the subheadings, i.e. treeline vegetations, treeline shift, tree regeneration dynamics of Himalayan alpine treeline, soil properties, growth and phenophase of treeline species. As the 5th step (interpretation and presentation), selected articles were considered and discussions were constructed to answer the research question. The network of keyword co-occurrences in the articles retrieved from the database search is displayed in Fig. 4. The network visualization highlights the connections between keyword nodes. Larger nodes in the lexical network represent keywords with higher frequencies of occurrence and links between nodes indicate that the corresponding keywords appear together in the same documents. Fig. 5 shows the number of articles published each year from 1986 to 2024. There is a gradual increase in annual publications, with notable peaks in 2018, 2020, 2022, and 2023, indicating a growing trend in recent years. Most of these studies were conducted using remote sensing, followed by field surveys, and focused on keywords such as vegetation shifts due to climate change, tree composition, plant diversity and regeneration status.

Based on the numbers of individuals of seedlings, saplings and trees, reported at different regions of the Himalayan alpine treeline, the regeneration status of tree species is classified (as outlined below) as Good, Fair, Poor, None and New [[50], [51], [52]].

**Table:** 

**Good**	**:**	seedling > sapling > adult
**Fair**	**:**	seedling > sapling ≤ adult
**Poor**	**:**	if a species survives only in the sapling stage but not as seedling (though sapling may be less, more or equal to adults)
**None**	**:**	if a species is absent in both seedling and sapling stages, but present as adults
**New**	**:**	if a species has no adults, but only saplings and/or seedlings present

## 3 Results and discussions

### 3.1 Vegetation diversity of the himalayan alpine treeline

The Himalayan alpine treeline occupies a vast territory, extending from Afghanistan in the northwest to Yunnan in the southeast [40]. It delineates a crucial ecological habitat between the forested area and the high-altitude alpine zones, providing a home for unique flora and fauna specifically adapted to the extreme conditions of this environment [[53], [54], [55]]. It is a climate-sensitive ecosystem and serves as a significant indicator of ecology for evaluating how the landscape responds to climate change [56,57]. This response is largely impacted by a combination of factors such as temperature, precipitation, soil conditions, and exposure to wind. The exact elevation of the treeline varies depending on the specific locations and the prevailing environmental conditions. Above this elevation, the harsh alpine conditions including cold temperatures, strong winds, and soil conditions significantly obstacles to the tree growth and rocky outcrops and snow defining the landscape. The Himalayan treeline supports a vast range of woody and non-woody plant species that represent extensive tree density and basal area. The vitality of this ecosystem depends on various anthropogenic and ecological factors, influencing species composition and overall structure [58]. Additionally, the Himalayan alpine treeline is a vital habitat for various animal species, including the snow leopard, musk deer, Himalayan black bear, etc. The surrounding area near this zone also provides important habitats for various wildlife, including birds and mammals. Padalia et al. [59] classified subalpine and alpine vegetation into 10 growth form classes, i.e. alpine meadows, alpine scrubs, shrubs/scrubs, broad-leaved deciduous forests, broad-leaved evergreen forests, needle-leaved forests, hill or dwarf bamboo, upper temperate grasslands, other vegetated surfaces and non-vegetated surfaces. The subnival zone is characterized by sparse vegetation located between the uppermost matted vegetation communities (primarily *Kobresia* sedge meadows and cushionoid vegetation) and the summer or permanent snow line. Three dominant alpine moist meadow classes were identified, i.e. mixed herbaceous meadows, *Kobresia*-dominated vegetation, and *Danthonia*-dominated vegetation. Alpine moist scrub classes are dominated by *R. anthopogon* and *Juniperus* species, which forms dense, dwarf, matted vegetation. Stunted woody vegetation, including krummholz and deciduous scrubs, covers extensive areas along the treeline-alpine ecotone. In certain locations, species of *Sorbus* and *Viburnum* are also found coexisting with *R. campanulatum*. Twelve subalpine vegetation classes were identified belonging to three broad physiognomic categories including subalpine broad-leaved evergreen (*Q. semecarpifolia-*dominated), subalpine broad-leaved deciduous (*Betula utilis* pure forest and mixed stands with *Acer* species), and subalpine needle leaves (*Abies pindrow, A. spectabilis, Pinus gerardiana, P. wallichiana, Cedrus deodara, Picea smithiana, Tsuga dumosa, Taxus wallichiana,* and *Cupressus torulosa*). The vegetation in the upper limit of the Himalaya acts as a natural buffer against soil erosion and landslides which is crucial for mitigating the potentially catastrophic effects of such events in the mountainous terrain, while also offering various ecosystem services [47,60].

Worldwide, pines are most strongly represented in treelines with 20 *Pinus* species occurring in these regions. Notable among them are *Pinus wallichiana*, *P. gerardiana*, *Abies*, *Picea* and *Larix* species found in the treelines of the Himalayas. Owing to their characteristics such as drought tolerance, fire resistance, ability to grow on nutrient-poor soils, shade intolerance, wind-dispersed seeds, and long lifespan [61,62], many pine species found in the Himalayas are well-adapted to the harsh conditions of treeline environments. The high species richness in the Himalayas can be partly attributed to the low tree limits, which are located at elevations lower than the climatic tree line. This phenomenon allows species from lower elevations, such as *Quercus semecarpifolia*, *Rhododendron arboreum* and *P. wallichiana*, to coexist at the treeline [63]. Many of the tree species in the Himalayan alpine treeline are endemic and are hardly mentioned in the literature. Mohapatra et al. [64] recorded the maximum biodiversity (Shannon's entropy, 3.04) in the 4000–4500 m elevation range along the eastern Himalayan alpine treeline ecotone. Wani et al. [65] reported 117 endemic and 88 threatened species in the tree database for the Indian Himalayan region. However, according to Tiwari et al. [66], alpine areas have a relatively smaller number of endemic plants. Globally, it is estimated that the number of tree species at the treeline does not exceed 100 species from 20 families [33]. Singh et al. [63] recorded a total of 58 tree species in the ecotones of the Himalayan timberline, spreading over 14 genera and 8 families. Sekar et al. [67] reported 265 vascular plants belonging to 155 genera and 55 families in Uttarakhand's alpine region (elevation 3200–4800 m). The majority of them were herbs (212), followed by shrubs (44) and trees (9). Among these, nine species were gymnosperms distributed across six genera and three families, while the remaining species were angiosperms. Khuroo et al. [35] documented 428 herbaceous species, 79 shrubs, 69 tree species, and 17 species that can grow both as shrubs and trees in the treeline ecotone of the entire Himalayas. In the timberline zone of the Kumaun Himalayas, Rawal and Dhar [68] identified 465 species with 64 % of them being indigenous to the Himalayas and more than one-third of the timberline flora are susceptible to endangerment, with 34 taxa requiring top-priority conservation at the local level, and 13 needing priority attention across the Himalayan region. The Himalayan treeline region harbours a rich diversity of plant species, including several of them are classified as threatened endemics due to habitat loss, climate change, overgrazing, and other anthropogenic pressures. Khuroo et al. [35] reported that in the Himalayan treeline ecotone flora, 2 plant species are currently recognized by the IUCN (International Union for Conservation of Nature) as Near Threatened, 4 as vulnerable, 7 as endangered and 2 as critically endangered. Salick et al. [69] reported a total of 762 taxa in the Himalayan alpine ecotone, of which 373 (48.95 %) were endemic. Maletha et al. [70] evaluated the threat levels of various tree species in the Himalayan alpine treeline region by assigning scores based on susceptibility and conservation priority. Species in Category I include *Betula utilis*, identified as the most vulnerable species at the timberline ecotone with a threat score of 29. Other vulnerable species were *Taxus baccata* (threat score: 26) and *Juniperus communis* (threat score: 25). Species with moderate threat levels were *Acer caesium*, *A*. *indica*, *Abies pindrow*, *Picea smithiana*, *Quercus torulosa* and *Rhododendron campanulatum*. Meanwhile, species in the vulnerable Category IV included *Cedrus deodara*, *Populus ciliata*, *Pinus wallichiana*, *Sorbus foliolosa*, *Salix sikkimensis* and *Viburnum grandiflorum*. Nine species had threat scores of 15 or higher, indicating that they may fall into the vulnerable category in near future. Negi et al. [71] reported a total of 409 taxa of angiosperms, gymnosperms and pteridophytes which are belonging to 203 genera and 71 families from the Nanda Devi National Park, which encompasses the Greater and Trans-Himalayan regions of Uttarakhand. Among these, 354 are herbs, 50 are shrubs, and 5 are trees (including *Betula utilis*, *Salix disperma*, *Acer acuminatum*, *Abies spectabilis*, and *Pinus wallichiana*). The study area also harbours a significant number of threatened and high-value medicinal species with high population densities. Out of the 409 recorded taxa, 24 plant species were classified as threatened according to the IUCN (2015). These include *Arnebia euchroma*, *Angelica glauca*, *Arnebia benthamii*, *Nardostachys jatamansi*, and *Cypripedium elegans*. Additionally, tree species such as *A. spectabilis*, *B. utilis*, and *P. wallichiana*, along with shrubs like *Juniperus communis*, *J. indica*, and *J. recurva*, were also listed under the threatened category. Mehta et al. [72] documented 456 threatened taxa in the Indian Himalayan region, spanning 233 genera and 87 families under various IUCN threat categories. Of these, 64 species fall into higher-risk categories including Critically Endangered (13), Endangered (28), and Vulnerable (23). Regionally, Sikkim reported the highest richness of threatened plants (203 species), followed by Himachal Pradesh (190), Jammu & Kashmir (189), Manipur, Arunachal Pradesh (147), and Uttarakhand (124). Anthropogenic activities like habitat loss, overgrazing, deforestation, over-exploitation, and unsustainable harvesting were major threats. Bisht et al. [73] reported higher plant species richness and diversity in the undisturbed alpine zones of the western Himalayas, specifically at the Valley of Flowers National Park and the Great Himalayan National Park, which recorded 155 and 215 taxa, respectively. These undisturbed sites exhibited greater biodiversity compared to the areas affected by tourism, trampling, over-exploitation etc. They also supported a significantly larger number of endemic and threatened species. In contrast, disturbed areas facilitated the proliferation of invasive species, including *Polygonum polystachyum*, *Rumex nepalensis*, *Impatiens sulcata*, *Urtica dioica* etc.

Nanda and Reshi [74] reported 235 plant species belonging to 168 genera and 71 families within the treeline ecotone of the northwestern Himalayas. Singh et al. [75] studied the plant diversity at the treeline ecotone in upper Bhaderwah of Jammu and Kashmir and found 253 species spanning 62 families, which includes 11 trees, 29 shrubs and 213 herbs with 247 angiosperms and 6 gymnosperms. Hamid et al. [76] documented 142 species from Kashmir Himalaya's alpine summits, spanning 116 genera and 38 families. In the Uttarakhand Himalayan alpine treeline region, Rawal et al. [77] reported 16 trees, 64 shrubs and 539 herbs species between 3000 and 3500 m elevation ranges. Along the Himalayan arboreal boundary *Abies*, *Betula* and *Rhododendron* species are the main tree species on the north-facing aspect while *Juniperus* sp. are the principal species on south-facing slopes [40]. Shrestha et al. [78] reported that in the central Himalayas, a dry south-facing slope is dominated by *Pinus wallichiana* and a moist north-facing slope is prevailed by *Abies spectabilis*. Mathew et al. [79] also observed a strong negative correlation (r = −0.96) between elevation and canopy height in the Himalayan alpine treeline ecotone. Gulzar et al. [80] recorded a total of 97 plant species belonging to 81 genera in 33 families at Khilanmarg and Sinthan Top treeline ecotone sites in Kashmir Himalaya. Bajpai et al. [81] reported 732 lichen species belonging to 148 genera and 47 families in the alpine zone of India. Sekar et al. [82] documented a total of 932 taxa, including 908 species, 5 subspecies, and 19 varieties, distributed across 371 genera and 76 families of angiosperms and gymnosperms. Among these, 797 are herbaceous species, 107 are shrubs, 22 are trees, and 6 are climbers, highlighting the rich floral diversity of the high-altitude cold desert region of Uttarakhand.

### 3.2 Impact of climate change on the himalayan alpine treeline

The influence of climate and topography on timberline and treeline are similar [83] multifaced and positively related to elevation [84]. Climate changes affect glaciers, snow, vegetation, and soils, have a significant impact on the alpine ecosystem and are very susceptible to them [85,86]. However, the impact of rising temperatures on the Himalayan treeline is complex and can vary depending on various factors such as the degree of warming, precipitation patterns, and other climatic variables. The rapid change in land use across the Himalayas stands is the most dominating factor in the abrupt temperature surge [40]. This high-altitude warming has effects on ecosystem functions and economic activity both directly and indirectly. Climate change has also led to an increase in extreme weather events, such as floods, landslides, and avalanches, in the Himalayan region. The warmer climate supports the growth of dwarf plant species like herbs and cushion plants [87]. Additionally, it leads to the retreat of the snowline and creates more favorable conditions for tree and plant growth at higher elevations. Consequently, this shift has prompted the upward migration of the treeline, which ultimately expanding grasslands and shrublands at higher elevations [40,88]. Salick et al. [69] highlighted the critical role of elevation and precipitation in shaping Himalayan alpine vegetation, and supports the predictions that climate change-induced rise in temperature and precipitation will have a significant impact on Himalayan biodiversity.

Researchers have unveiled that the alpine tree belt borderline has been affected by a range of climate change events, such as global warming, landslides, and floods, resulting in soil erosion and vegetation loss [6,89]. For instance, changes in precipitation patterns can have a significant impact on the treeline, as it plays a critical role in determining the water availability for plants [37,90]. Additionally, the melting of glaciers and snow has also resulted in increased water availability in the region [91], which has affected the soil and plant communities in the arboreal boundary. Bharti et al. [88] reported that the loss of mountain snow cover (between 1998 and 2010) due to climate change and global warming increased the forest area, krummholz, alpine scrub, alpine meadow, and bare land in the alpine region of Uttarakhand Himalaya. Early snowmelt, driven by rising temperatures, has been also associated with increased diversity and density within herb communities [92]. This shift in snowmelt timing significantly impacts alpine plant species' growth and viability [93]. The altered snowmelt patterns can create more or less suitable areas for plant growth. These changes can lead to shifts in alpine plant communities, favouring the dominance of warm-adapted species and potentially resulting in the loss of cold-adapted species, a phenomenon known as thermophilization [94,95]. Adhikari et al. [92] reported 86 herb species and examined that early snowmelt treeline sites exhibit higher species richness and diversity compared to late snowmelt sites, across high and low snow cover areas. Additionally, plant density ranged from 82 to 626 individuals m^−2^ in early snowmelt sites and 69 to 288 individuals m^−2^ in late snowmelt sites ecotone in Uttarakhand Himalaya. Singh et al. [7] reported that early snowmelt boosts *R. campanulatum* elevation and enhances plant diversity. Kuniyal et al. [96] also reported *Rumex nepalensis* (an invasive herb) in the treeline zone of Uttarakhand, which may be considered an indicator of plant invasion threat in this unique ecosystem.

Wang et al. [97] reported that alpine tree limits exhibit different responses to climate warming, with varied responses despite overall increases in tree growth and recruitment. While many treelines moved forward, others stayed unchanged. The expansion potential index positively correlated with summer warming rates and treeline shifts, emphasizing the importance of current locations of tree species in comprehending treeline changes amidst climate change on the Tibetan Plateau. Huang et al. [98] studied 11,804 treeline locations in the eastern Himalayas and reported that treeline elevation is positively linked to summer temperature and snowline elevation, but negatively correlated with snow cover days and spring temperature. Notably, spring snowline elevation (33.4 %) and seasonal snow cover days (21.1 %) were the most influential factors in determining treeline elevation, surpassing permanent snowline, temperature, precipitation and light. In this context, climate change refers to the evolving conditions along the central Himalayan tree frontier line, characterized by diminished cold temperatures and reduced constraints on spring precipitation. Tree ring data from 1858 to 2015 for *A. spectabilis* and 1805 to 2015 for *B. utilis*, indicates that *A. spectabilis* is more responsive to summer temperatures, whereas *B. utilis* is influenced by pre-monsoon precipitation [99]. Singh et al. [100] reported that air temperatures have risen by 0.3 °C per decade in Sikkim Himalaya, benefiting treeline growth in temperature-constrained ecosystems. Meanwhile, annual precipitation has dropped by 206.50 mm per decade. Timberline under low temperatures and growth limitation, is the primary physiological factor, while, scarcity and water stress don't directly induce timberline formation [101]. The various effects of climate change on Himalayan treeline species through physiological, morphological, and phenological traits, are presented in Table 1. Most alpine treeline species adapt to climate change by migrating upward, and phenological changes are also reported due to climate change and global warming. For instance, *A. pindrow* demonstrates a connection between carbon-bound hydrogen and cellulose with the growing season and climate parameters, while *A. spectabilis* shows a positive growth trend at the treeline influenced by a warming climate. *Betula utilis* and *Q. semecarpifolia* exhibit phenological variations related to temperature and moisture, whereas *Rhododendron* species display changes in morphology and phenology influenced by water stress and temperature fluctuations. The multifaceted impacts of climate change and global warming are discussed under the following subheadings.

**Table 1:** Climate-related physiological, morphological and phenological responses in the Himalayan alpine treeline species.

Species	Physiological	Morphological	Phenological	References
*Abies pindrow*	Carbon-bound hydrogen & cellulose show a significant relation to growing season climate parameters.	Tree rings as proxies extend climate data; positive correlation with summer precipitation and temperature.	Shifts in phenology observed; Growing season decreases by 11.2 days per °C or 4.5 days per 100 m elevation increase.	[[102], [103], [104]]
*Abies spectabilis*	Osmotic potential changes at full and zero turgor.	Positive growth trend at treeline due to warming climate; limited by low temperatures and spring moisture availability.	Climate warming causes moisture stress; phenophase variations significantly correlate with mean annual temperature.	[31,[105], [106], [107]]
*Betula utilis*	Seasonal osmotic potential changes; Twig pressure potential and leaf conductance rates exhibit opposite seasonal trends.	–	Phenological phases are influenced by temperature and day length.	[31,105,108]
*Quercus semecarpifolia*	Changes in osmotic potential across seasons.	–	Phenophase variations significantly correlate with temperature and rainfall; delays in leaf emergence, damage, and senescence.	[31,105]
*Rhododendron arboreum*	Floral morphology is adapted for bird pollination	Distribution is influenced by annual precipitation and temperature.	Early phenophase and reproduction phenology stages; flowering triggered by water potential threshold; leaf and bud size correlated with twig water potential and soil moisture.	[31,[109], [110], [111], [112]]
*Rhododendron campanulatum*	Seasonal osmotic potential changes; increased photosynthesis at higher temperatures.	Reduced height & leaf size due to water stress; leaf traits vary with elevation (increased thickness, decreased length & width).	Phenophase variations significantly correlate with mean annual temperature.	[31,105,113]
*Rhododendron lepidotum*	–	Morphological and anatomical features vary along the elevation gradient, suggesting climate adaptation.	–	[114]

#### 3.2.1 Treeline shift

The upper tree limit of Himalayan ecosystems is particularly vulnerable to warming [13], leading to anticipated species relocations or habitat shifts due to climate change [115]. However, the response to climate warming over the last century has been mixed, with some treelines showing evidence of recruitment at higher altitudes or latitudes, while others show no discernible shift in the upper limit of tree establishment [116]. Contemporary climate warming is considered the key driver of recent shifts in alpine plant distributions [94,95]. Rising temperatures are causing the treeline to shift upwards, and the frequency and intensity of extreme weather events are increasing [39,117,118]. Treeline advance shifts of up to 70–100 m have been reported from several Northern Hemisphere mountain ranges and attributed to climate change [40,[119], [120], [121]]. According to Gaire et al. [122], *A. spectabilis* trees at all elevations may benefit from rising summer temperatures if there is adequate moisture. Furthermore, winter warming may benefit trees further at the upper treeline. Harsch et al. [116] investigated a global dataset of 166 locations where treeline dynamics had been documented since 1900 AD to explore variation in the treeline. Out of 166 sites, 87 sites (52 %) demontrated treeline advancement, only 1 % treeline recession and 77 sites (47 %) had remained stable or did not show elevational shifts. This shift has been observed in several regions, including the Himalayas, where the treeline has increased by an average of 30–40 m over the past few decades (Table 2). According to the dendroecological analysis conducted by Khandu et al. [123], it was reported that the diffuse treeline ecotones in Northern Bhutan are advancing at an average rate of 1.00 m year^−1^. Maharjan et al. [124], using species distribution models for 1985 and 2050 conditions, predicted an average upward shift of the treeline by 3 m per year in optimal elevation, with a 33 % increase in distribution area in the Nepal Himalaya. Rather than an upward shift of treeline, Singh et al. [38] and Sah et al. [125] also reported a downward movement of 56 m between the years 1977–2015 of the Himalayan alpine treeline in Sikkim Himalaya. This phenomenon is particularly intriguing as it contrasts with the generally observed upward movement of treelines in response to global warming. This downward shift could be due to a complex interplay of edaphic and climatic variations. The topography and physicochemical properties of the soil may have influenced the ability of the tree line to maintain vegetation at higher elevations, resulting in a regression of its position. In addition, climatic fluctuations, including changes in temperature, precipitation patterns and seasonal weather events, may have created unfavourable conditions for tree regeneration and growth at the former treeline, contributing to its downward shift. This observation underscores the importance of considering both local environmental conditions and broader climate trends when analysing the dynamics of the tree line in mountain regions. Remote sensing analysis shows no altitudinal change in the upper treeline over 40 years, despite significant canopy cover fluctuations in the treeline areas of western Himalaya [126]. The shift in the treeline boundary has resulted in the loss of habitat for several alpine plant and animal species, which are adapted to the harsh conditions of the timberline [127,128]. This could affect the area of forests in the Himalayan region and potentially lead to changes in the ecosystem dynamics. Hamid et al. [76] found changes in species richness, vegetation cover, and soil temperature across an alpine elevation gradient from 2014 to 2018. Sekar et al. [129] conducted a study following the Global Observation Research Initiative in Alpine Environments (GLORIA) protocol to evaluate the temporal dynamics of vegetation on alpine and nival summits in the western Himalaya. The summits, initially established in 2014–15, were surveyed to record baseline vegetation data and resurveyed five years later. The study reported a significant increase in mean species richness (6.3 %) and vegetation cover (13 %) between the baseline and resurvey datasets. Additionally, a decrease in nestedness and a substantial increase in species turnover were observed, highlighting species replacement as the primary driver of temporal variation in β-diversity. These changes suggest potential shifts in the composition and structure of plant communities in the near future in alpine regions.

**Table 2:** The average upward shift rates of the alpine treeline or timberline.

Region		The upward shift of Himalayan treeline or vegetation	Time (in years)	Reference
Western Himalaya		300 m	44 (1960–2004)	[117]
Gangotri glacier area, Western Himalaya		327 ± 80 m	30 (1976–2006)	[130]
Uttarakhand Himalaya, India		388 ± 80 m	36 (1970–2006)	[39]
Uttarkashi, Uttarakhand, India		360 m		
Tehri Garhwal, Uttarakhand, India		400 m		
Rudrapyag, Uttarakhand, India		390 m		
Chamoli, Uttarakhand, India		430 m		
Bageshwar, Uttarakhand, India		360 m		
Pithoragarh, Uttarakhand, India		390 m		
Barun valley, Eastern Nepal Himalaya		22 ± 5.5 m	130	[131]
Yamunotri watershed, Garhwal Himalaya		80 m	20 (1990–2010)	[132]
Qilian Mountains, Tibetan Plateau		51.5–79.9 m	80 (1923–2003)	[133]
Wulan, Tibetan Plateau		13.2–53.6 m		
Sygera Mountains, Tibetan Plateau		0–0.80 m		
Ranwu Lake, Tibetan Plateau		3.6–68.5 m		
Baima Snow Mountains, Tibetan Plateau		18.7–28.1 m		
Central Himalaya (Nepal)		16.5 m	150	[118]
Sikkim Himalaya		301 ± 66 m	37 (1977–2013)	[100]
Arunachal Pradesh, India	East aspect	430 m	41 (1973–2014)	[134]
	West aspect	503 m		
	North aspect	486 m		
	South aspect	420 m		
	Mean upward shift	452 ± 74 m		
Himalayan region of Arunachal Pradesh		452 ± 74 m	40 (1974–2014)	[135]
Pinder Watershed, Uttarakhand Himalaya		166 m	29 (1990–2019)	[136]
Jammu & Kashmir, India		441 ± 71 m	42 (1972–2014)	[137]
Himachal Pradesh, India		301 ± 77 m		
Uttarakhand, India		411 ± 79 m		
Sikkim, India		301 ± 66 m		
Arunachal Pradesh, India		452 ± 74 m		
Astore and Bagrote Valley of Pakistan	Astore valley–1	41.30 m	407 (1611–2018)	[138]
	Astore valley–2	6.80 m	257 (1761–2018)	
	Astore valley–3	63.0 m	407 (1611–2018)	
	Bagrote valley–1	16.50 m	307 (1711–2018)	
	Bagrote valley–2	3.50 m	307 (1711–2018)	
Kashmir to Bhutan Himalaya		7 to 28 ± 1.5 m yr^−1^	14 (2000–2014)	[139]
Sikkim Himalaya, India		100 m	38 (1977–2015)	[38,125]

The competitive advantage of *R. campanulatum* in forming dense krummholz belts due to climate change may clog or hold up the upward migration of tree species in the Himalayan treeline, thus affecting response to climate change [[140], [141], [142]]. This could be attributed to its high competitiveness, absolute dominance, and allelopathic effects, as suggested by various studies [40,115,142]. Singh et al. [143] found that the densification of treeline ecotone and the advance of krummholz species (*R. campanulatum*) into the alpine meadows of the Himalayas pose serious threats to diversity and integrity. This increase in woody cover can lead to reduced albedo and daytime soil cooling. Additionally, the upward movement of *R. campanulatum* due to climate warming may deplete alpine meadows as it prefers a cool and moist climate [144]. In contrast, winter warming seems to favour *A. spectabilis*, with early snowmelt increasing growth period and species richness. Treeline vegetation responds differently to environmental factors, with both juvenile and adult trees affected to varying degrees. *R. campanulatum* thrives in the krummholz belt, shaped by specific site conditions, while *A. spectabilis* and *B. utilis* dominate warmer, nutrient-rich habitats in lower-closed forests. *A. spectabilis* growth is influenced by changing climate conditions, particularly decreasing moisture availability before the monsoon season, which impacts radial growth. Mainali et al. [145] found that *A. spectabilis* thrives in deep shade, while *R. campanulatum* needs sunlight for seedling growth. Latwal et al. [84] reported patches of different sizes were formed by *R. campanulatum* mixed with a few individuals of *Abies*, *Betula*, and *Sorbus* in Uttarakhand Himalaya. As *R. campanulatum* extends above the treeline, it shades out ground vegetation, facilitating *A. spectabilis* establishment. This dynamic suggests potential future treeline shifts, with *R. campanulatum* likely dominating above treeline in the coming decades. Singh et al. [141] estimated 1.4 m year^−1^ expansion rate of the *R. campanulatum* population. This growth suggests the dominance of *R. campanulatum* over other tree species like *A. spectabilis*, *B. utilis*, and *Q*. *semecarpifolia* due to its nonpalatable nature, potentially impacting the treeline ecosystem. Wang et al. [146] observed a generally positive effect of global warming on tree growth across both canopies, and most species projecting average growth gains of 7.8 %–12.2 % due to climate change. Karki et al. [147] reported that *R. campanulatum* was regenerating effectively, with stronger regeneration observed at higher elevations (3800 m asl) compared to lower elevations. The study revealed that tree canopy cover largely determines the extent of regeneration, as evidenced by the greater number of seedlings observed in treeless stands. Despite climate-induced changes in stand densities and growth patterns, the treeline position remains stable for now, with a potential shift anticipated in the mid-to-long term [148]. The sensitivity of tree species to climate change varies depending on their ecological niche and their evolutionary history. For example, species that have evolved in stable environments may have difficulty adapting to rapid change, while species with greater phenotypic plasticity may thrive under new conditions [149]. Plants can also adapt to changing climates through epigenetic mechanisms, which allow for rapid adjustments in gene expression without altering the DNA sequence. This plasticity enables trees to respond to environmental stimuli, such as changes in temperature and precipitation, which are critical for their survival and distribution [150,151]. Genetic diversity within tree species plays a crucial role in their ability to adapt to climate change. Populations with higher genetic variability are more likely to have individuals that can withstand environmental stressors, leading to better overall resilience of the species [151]. The responses of tree species to climate change are complex and are influenced by a combination of physiological, ecological and genetic mechanisms. The observed differences between species can be attributed to their unique adaptations, growth patterns and evolutionary histories, which determine their resilience or vulnerability to climate-induced changes.

Several studies reported the upward shift of alpine treeline species, of which *A. spectabilis* and *B. utilis* are the most sensitive tree species in the Himalayan treeline regions (Table 3). Panthi et al. [152] suggest that future winter warming may benefit *R. campanulatum* growth and promote upslope expansion of alpine vegetation. Singh et al. [153] also observed *R. campanulatum* seedlings were present 6–20 m beyond the krummholz and showed an upward movement from the actual treeline limit encroaching into alpine meadows. Gaire et al. [154] reported a decline in the long-term growth trends of *A. spectabilis* at low-to mid-elevation locations, while an increase was observed in the treeline zone of Mount Everest between 1981 and 2011 due to climate change. Schickhoff et al. [40] observed a significant variation in bioclimatic variables, it is expected that the possible niche of *B. utilis* migrate from lower to higher altitudes and expand into new habitats, i.e. north of the Himalayan range. Manish and Pandit [155] found that the majority of plant species prefer mid and higher elevations under the current climatic conditions (2000–4500 m), and almost all of these plant species show a northward shift from their possible habitats towards higher elevations in future climates, regardless of pollution scenarios. Singh et al. [130] reported that the alpine treeline of the Gangotri glacier area (Uttarakhand Himalaya) is expected to go further up to 5914 m in 2080 compared to 5347 m in 2010.

**Table 3:** Upward shift rate of Himalayan treeline species.

Region		Species	Upward shift rate (m yr^−1^)	Reference
Himachal Pradesh, Western Himalaya	South aspect	*Pinus wallichiana*	1.90	[156]
	North aspect		1.40	
Manaslu Conservation Area, Nepal Himalaya		*Abies spectabilis*	3.40	[157]
Gangotri glacier area, Western Himalaya		*Betula utilis*	3.00	[130]
Manaslu conservation, Central Nepal Himalaya		*Abies spectabilis*	2.61	[89]
Barun valley, Makalu National Park, Eastern Nepal Himalaya	South aspect	*Abies spectabilis*	0.122–0.161	[131]
	North aspect		0.00–0.222	
	East aspect		0.301	
Chimkhola area, Myagdi district, Nepal	South-east aspect	*Abies spectabilis*	1.36	[158]
	North-west aspect		0.56	
Chimang Lekh, Mustang District, Nepal	Chimang 1	*Abies spectabilis*	0.50	[159]
	Chimang 2		2.21	
Sagarmatha (Mt. Everest) National Park, Nepal		*Abies spectabilis*	0.93	[160]
		*Betula utilis*	0.42	
Uttarakhand, Himachal Pradesh		*Pinus wallichiana*	1.10–5.40	[62]
Chopta, Tungnath, Uttarakhand		*Abies spectabilis*	1.31	[161]
Central Himalaya	Kanchanjunga	*Betula utilis*	0.20–0.37	[118]
	Everest	*Abies spectabilis*	0.10–0.34	
	Langtng	*Betula utilis*	0.08–0.17	
	Manag	*Abies spectabilis*	0.07	
		*Betula utilis*	0.03–0.05	
	Jumla	*Betula utilis*	0.04	
	Humla	*Abies spectabilis*	0.02–0.03	
		*Betula utilis*	0.10–0.11	
Jammu & Kashmir, India		*Betula utilis*	2.10	[137]
Himachal Pradesh, India			4.60	
Uttarakhand, India			7.80	
Sikkim, India			11.00	
Arunachal Pradesh, India			4.50	
Lahaul valley, North-western Himalaya		*Juniperus macropoda*	3.91	[162]
Tungnath, Uttarakhand		*Abies spectabilis*	1.00	[163]
Sagarmatha National Park and Annapurna Conservation Area, Nepal Himalayan treeline		*Abies spectabilis*	0.11	[164]
		*Betula utilis*	0.06	

Climate change has caused a shift in the growth sensitivity of *A. spectabilis* at a central Himalayan treeline ecotone [165]. The presence of only small-sized trees with strong recent regeneration of *A. spectabilis* at the upper treeline suggests densification and potential upward migration in response to environmental shifts, including climate change [166]. *Abies spectabilis* in Nepal Himalaya show rapid stand densification and shifting rates, contrasting with the slow changes observed in *B. utilis* [41]. Tiwari et al. [159] found that the sub-alpine treeline of *A. spectabilis* is shifting upward due to increased seedling recruitment and growth attributed to rising temperatures, snowmelt and precipitation. Chhetri et al. [167] predict that by 2050–2070, *A. spectabilis, B. utilis* and *P. wallichiana* will migrate upwards in the Nepal Himalayas due to climate change. Tiwari et al. [42] found varying treeline positions and tree species limits in the Nepal Himalayas, notably *A. spectabilis* seedlings at 38 m above the treeline. *A. spectabilis* and *B. utilis* treelines shifted consistently with abundant seedling recruitment, while, *A. georgei* and *Larix potaninii* treelines showed lower shifting rates and seedling recruitment. Range shifts varied from 0.11 to 1.74 m year^−1^, with some sites showing centuries-long changes in treeline position. The statistics and comparisons confirmed that the tree rings of *Abies densa* at the tree lines of Northern Bhutan reflect temperature sensitivity due to global warming [168]. Liang et al. [169] found little change in the *Abies georgei* tree line position in the Tibetan plateau after 200 years of warming. Mohapatra et al. [135] predicted that *B. utilis* is vulnerable to 21st century climate changes in the Hindu Kush Himalaya. Yadava et al. [62] found that the *P. wallichiana* treeline in the western Himalayas has shifted upward, with rates varying from 11 to 54 m decade^−1^, influenced by microclimatic and biotic factors. Misra et al. [170] reported the presence of juvenile saplings of *Juniperus polycarpos* above the treeline in the Kashmir Himalayas.

Climate change is the most influential driver altering species' natural habitats, resulting in global biodiversity loss [171]. Some plant and animal species can adapt and shift due to the impact of climate change and global warming, while others could speed its demise or extinct. Treeline vegetation has been severely miserable as a result of biotic and abiotic strains over time in the Himalayas [172]. Sigdel et al. [164] investigated the interactions and successional strategies of the early-successional *Betula utilis* and the late-successional *Abies spectabilis* to understand their impact on treeline dynamics in the central Himalayas. Spatial analysis highlighted intense interspecies competition among young trees. Climate models suggest that *A. spectabilis* is likely to shift further upslope with warming temperatures, while *B. utilis* recruitment may decline, potentially stabilizing or causing a retreat of the treeline. Overall their findings revealed that both species (*B*. *utilis* and *A*. *spectabilis*) have experienced increased recruitment and a higher rate of upslope shifts over the past 200 years due to climate change. The potential distribution modelling of *Taxus wallichiana*, *Dactylorhiza hatagirea* and *Paris polyphylla* in the Nepal Himalaya suggests that the habitat degradation is occurring as a consequence of weather pattern shifts [172,173]. Dhyani et al. [171] reported potential habitats of *Hippophae salicifolia* are rapidly declining in the central Himalayas and projected that 87.2 % of the suitable areas for this species will be lost by 2050. Additionally, an upwards shift in the species' habitat by 1700 m above mean sea level (amsl) to the range of 2800–4500 m amsl is also predicted based on the potential habitats of *H*. *salicifolia*. Sekar et al. [174] also observed a declining trend in the population of *Juniperus semigloboosa* in the alpine region of Uttarakhand. Climate warming is projected to increase species accumulation near the treeline due to global warming. This is facilitated by the existence of vast continuous timberline which provide ample land between the snowline and the timberline for plant migration [175]. Hamid et al. [176] used species distribution modelling to forecast the shifting bioclimatic ranges of *B. utilis* in the Himalayas due to climate change. Their study suggests that the Western Himalayas, including northern Pakistan and northwest India, will likely have ideal climatic conditions for *B. utilis*.

Baniya et al. [177] found significant NDVI (Normalized Difference Vegetation Index) increases in Langtang National Park (0.002 yr^−1^) and its treeline ecotone (0.003 yr^−1^) from 2000 to 2017. The park covers 68.73 % (1463 km^2^), with 16.45 % (350.43 km^2^) greening and 0.25 % (5.43 km^2^) browning. NDVI in the treeline ecotone ranged from 0.1 to 0.5, with positive changes in vegetated areas and negative changes in barren lands. Wang et al. [178] gathered data on 128 endemic species from the eastern Himalayas, spanning 49 genera and 24 families, which are known to inhabit areas above the upper tree limit, to assess potential habitat loss due to upshifts. Panigrahy et al. [117] analyzed satellite data to track changes over time. In 1986, snow and glaciers covered 90.5 % of the Nanda Devi Biosphere Reserve, while scree accounted for 9.0 %, and vegetation only 0.5 %. By 1999, there was little change in snow and glaciers, but a slight increase in vegetation by 1.8 %. In 2004, significant changes occurred, i.e. scree dominated (42 %), followed by snow/glacier (35 %), and vegetation (23 %).

#### 3.2.2 Tree regeneration

The regeneration status of the species depends on a sufficient number of seedlings, saplings and trees. The transformation from seedlings to adult trees is important and therefore the regeneration dynamics is a major thrust area of the Himalayan alpine treeline area [153]. Climate change and global warming have a significant impact on this treeline regeneration [175]. Meanwhile, good or fair regeneration of treeline species indicates the species' upward shift [179]. Gaire et al. [106] found that site and species-specific regeneration sustain treeline dynamics, and accounted an average upward shift of 0.46 m year^−1^ in the Nepal Himalayas due to climate change factors like temperature and precipitation influence. Sharma et al. [113] reported a stem density of 3458 stems per hectare of *R*. *campanulatum* in the Mustang district of Nepal, comprising 1176 seedlings, 482 juveniles, and 1800 adults above the treeline. The high density of seedlings and saplings above the treeline ecotone, coupled with the high mortality of seedlings below the treeline, suggests robust regeneration of the species above the treeline. This pattern is consistent with the observed population growth of *R. campanulatum* in the treeline regions of the Nepal Himalayas. Gupta et al. [180] reported poor regeneration of *A. spectabilis* and low recruitment of seedlings and saplings at the treeline ecotone (3340 m elevation) in Tungnath, Uttarakhand Himalaya, in contrast to the higher recruitment of *R. campanulatum* in the meadow zone, where it forms a krummholz line at 3550 m elevation. Khandu et al. [123] reported successful tree regeneration of *Abies densa* seedlings thriving above adult tree limits. Schwab et al. [142] identified diverse transition patterns across the treeline ecotone that have significantly impacted the stand structure and are correlated with soil temperature and spatial regeneration. Despite the current stability observed, they suggest the potential for upslope migration among treeline species, highlighting prolific regeneration and stand densification. Research on seedling establishment and tree recruitment in treeline ecotones is limited, leading to a lack of understanding of tree recruitment patterns [40,115,181]. Studies mainly focused on stand age structure and population demography, with Himalaya's treelines showing promising regeneration trends but some areas exhibiting poor regeneration [41,53,89,113,182,183]. Frequent dry spells lasting 6–8 days after intermittent rainfall lead to seed mortality, evidenced by numerous seeds with dried and varying-length radicals (2–6 cm) [184]. Singh et al. [63,153] reported that poor to absent regeneration in *A. spectabilis*, *Acer caesium*, *B. utilis*, *Prunus cornuta*, *Q. semecarpifolia* and *Taxus baccata*; while *Rhododendron arboretum* (90–230 ind. ha^−1^) and *R. campanulatum* (190–330 ind. ha^−1^) showed the good regeneration status in western Himalayan treeline area. Gaire et al. [185] reported a spatially heterogeneous distribution of tree seedlings and saplings in the treeline areas of Langtang National Park in Central Nepal. The average seedling density for *A. spectabilis* was 330 seedlings ha^−1^, for *B. utilis* it was 20 seedlings ha^−1^, for *Juniperus recurva* it was 48 seedlings ha^−1^ and for *Sorbus microphylla* it was 45 seedlings ha^−1^. These values were higher than the saplings density for the respective species, which were 255 saplings ha^−1^, 37 saplings ha^−1^, 71 saplings ha^−1^ and 41 saplings ha^−1^, respectively. Furthermore, the saplings density of *R. campanulatum* was notably higher as 1186 saplings ha^−1^ compared to its seedling density of 368 seedlings ha^−1^, owing to its krummholz nature.

**Fig. 6:**
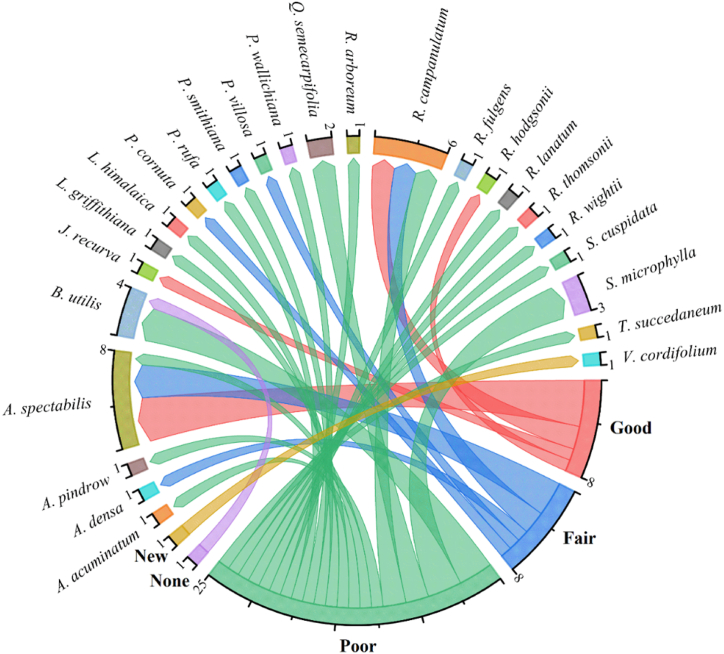
Chord diagram illustrating regeneration patterns of reported Himalayan alpine treeline species.

Schwab et al. [186,187] surveyed 8010 individuals of *A. spectabilis*, *B. utilis*, and *R. campanulatum* including 3486 seedlings, 3262 saplings and 1262 adult trees and predicted that the tree population densities accurately emphasizing soil, topography, climate, and species interactions. The study suggests climate change may trigger diverse, non-linear responses in tree populations and treeline positions [187]. In Rolwaling Valley of Nepal, Schickhoff et al. [115] found strong regeneration and growth of *B. utilis*, *A. spectabilis*, *R. campanulatum*, and *Sorbus microphylla* seedlings and saplings, even surpassing the usual treeline limit by 100–150 m. Burzle et al. [188] also found diverse seedling densities in Rolwaling Valley, comprising 784 *A. spectabilis*, 155 *B. utilis* and 1191 *R. campanulatum* seedlings per hectare along with high mortality rates during recruitment, and the distribution varied across vegetation types, underscoring uneven establishment. Schickhoff et al. [189] found robust regeneration of *B. utilis*, *A. spectabilis*, and *R. campulatum* above the adult tree line in the Rolwaling Himalaya, with some exceeding 2 m in height even beyond the 0–200 m of krummholz zone. Rana et al. [190] reported that *R. campanulatum* and *A. spectabilis* exhibited a sustainable regeneration pattern, whereas *B. utilis* and *S. microphylla* showed poor regeneration, possibly attributable to unfavourable microhabitats in the Manaslu conservation areas of Nepal. The diameter class of treeline vegetations showed a near inverse J-shaped distribution, suggesting sustainable regeneration [191] while a reverse J-shaped curve was observed by Rai et al. [126]. Rawal et al. [192] found a hill-shaped population structure, suggesting inadequate tree species regeneration. Forest patches in the Western Himalayas surveyed for treeline tree species regeneration displayed limited natural regrowth, especially outside protected areas. *B. utilis* showed abundant seedlings (1450–1860 individuals ha^−1^) and saplings (930–400 individuals ha^−1^) in minimally human-impacted areas, maintaining a near-natural population structure. While disturbed sites near *Q. semecarpifolia* timberlines exhibited a high abundance of seedlings, whereas the conversion rate to saplings was low. In Dhorpatan Hunting Reserve, Nepal, Chhetri and Cairns [193] reported no seedlings or saplings above the treeline region in transect A, while in transect B, the sapling observed was at 3895 m, coinciding with the species limit. Factors like moisture scarcity, inadequate microsites, and herbivore presence restrict seedling and sapling growth. The *B. utilis* treeline stands are relatively young, averaging 53 and 62 years in Transects A and B, respectively [193]. Chandra et al. [194] grew the selected seedlings of treeline plant species in open-top chambers where CO_2_ concentration was increased from ambient (400 ppm) to elevated (650 ppm) levels. They found increased assimilation rates and growth for *Acomastylis elata* and *Anaphalis nepalensis*, but decreased photosynthesis for *Bistorta macrophylla* and *Trillium govanianum*. Among the different species, *A. spectabilis* is strongly associated with fair regeneration, while *B. utilis*, *S. microphylla* and other species predominantly fall into the poor regeneration category. *R. campanulatum* exhibits a mixed regeneration pattern, with good, fair, and poor regeneration statuses. Additionally, *B. utilis* also shows instances of no regeneration, and *V. cordifolium* has been reported as a new species in the Himalayan treeline region. The majority of Himalayan treeline species are reported to have poor regeneration status, followed by fair and good regeneration. These varied outcomes across species suggest that diverse environmental or ecological factors are influencing regeneration in the alpine treeline of the Himalayas. Based on the number of individuals of seedlings, saplings and trees, Table 4 and Fig. 6 represents the regeneration status of different treeline tree species of the Himalayas.

**Table 4:** Regeneration status of different species of Himalayas alpine treeline region.

Location		Tree species	Plant life-form (individuals ha^−1^)			Regeneration status	Reference
			Seedlings	Saplings	Trees		
Dzongri region of Khangchendzonga National Park		*Abies densa*	121.20	35.58	63.56	Fair	[183]
		*Prunus rufa*	13.52	33.64	35.37	Poor	
		*Sorbus microphylla*	33.70	105.30	104.27	Poor	
		*Pieris villosa*	2.96	0	1.48	Fair	
		*Rhododendron arboreum*	1.48	3.58	4.07	Poor	
		*Rhododendron hodgsonii*	30.36	27.70	22.00	Good	
		*Rhododendron lanatum*	44.10	110.65	92.60	Poor	
		*Rhododendron wightii*	29.20	30.80	40.15	Poor	
		*Rhododendron fulgens*	2.22	15.80	5.93	Poor	
		*Rhododendron thomsonii*	1.85	2.22	05.00	Poor	
		*Viburnum cordifolium*	5.19	0	0	New	
Langtang National Park, Nepal		*Abies spectabilis*	350	255	261	Fair	[185]
		*Betula utilis*	20	37	56	Poor	
		*Juniperus recurva*	48	71	34	Good	
		*Rhododendron campanulatum*	368	1186	379	Fair	
		*Sorbus microphylla*	45	41	25	Poor	
Langtang National Park, Nepal		*Abies spectabilis*	350	255	236	Good	[182]
Kanchenjunga and Manaslu Conservation Area, Langtang National Park, Nepal		*Larix griffithiana*	73.5	206	253.5	Poor	[195]
		*Larix himalaica*	0	27	280	Poor	
Barun Valley, Nepal	South aspect	*Abies spectabilis*	555	116	144	Fair	[196]
	North aspect		455	90	101	Fair	
	East aspect		1400	169	113	Good	
Above treeline of Annapurna Conservation Area in Nepal		*Rhododendron campanulatum*	1176	482	1800	Fair	[113]
Khangsar Forest of Central Nepal		*Abies spectabilis*	1154	166	13	Good	[166]
Chattergalla pass to till lake Kailash timberline ecotone in Jammu & Kashmir Himalayas		*Abies pindrow*	26	39	63	Poor	[53]
		*Betula utilis*	20	45	61	Poor	
		*Acer acuminatum*	2	3	7	Poor	
		*Picea smithiana*	15	30	71	Poor	
		*Pinus wallichiana*	13	25	74	Poor	
		*Prunus cornuta*	3	4	4	Fair	
		*Quercus semecarpifolia*	31	93	214	Poor	
		*Sorbus microphylla*	2	6	11	Poor	
		*Sorbus cuspidata*	2	3	7	Poor	
		*Toxicodendron succedaneum*	0	1	7	Poor	
Tungnath treeline area, Uttarakhand		*Quercus semecarpifolia*	21	0	61	Poor	[184]
Manaslu Conservation Area, Nepal Himalaya	Transect 1	*Abies spectabilis*	254	62	84	Good	[89]
		*Betula utilis*	0	0	224	None	
	Transect 2	*Abies spectabilis*	8	42	36	Poor	
		*Betula utilis*	0	4	156	Poor	
Tungnath, Uttarakahnd Himalaya	3100 m elevation	*Rhododendron campanulatum*	700	415	2845	Poor	[179]
	3250 m elevation		980	600	2150	Poor	
	3400 m elevation		3480	2210	1840	Good	
	3450 m elevation		2360	1590	1210	Good

The data highlights the diverse regeneration statuses of tree species across different locations in the Himalayan alpine treeline region, emphasizing the intricate interactions between climate, soil conditions, and species-specific traits. As some areas face challenges in natural regeneration due to shifting climate patterns, while others may experience changes in species composition or forest density. This species consistently exhibits fair to good regeneration across different aspects and elevations, indicating its adaptability to changing climatic conditions. In contrast, *R. campanulatum* observed in the Annapurna conservation area [113] and Tungnath [179], shows considerable variation in regeneration status based on elevation. This species shows poor regeneration at lower elevations but improves significantly at higher elevations, where it dominates the alpine treeline zone [179]. Meanwhile, species such as *R. hodgsonii* and *A. densa* maintain a relatively stable regeneration status in the Dzongri region, indicating their resilience to current climatic conditions. Conversely, *Q. semecarpifolia*, *B. utilis*, *R. arboreum*, *P. rufa* and *S. microphylla* show poor regeneration across most locations, marking potential challenges in their regeneration processes. These challenges could be linked to factors such as competition, soil degradation, specialized habitat requirements, and changing climate conditions. The findings also highlight the impact of elevation and aspect on regeneration. In Barun Valley, significant differences in regeneration status are observed across various aspects, with the east aspect showing better regeneration for *A*. *spectabilis* compared to the north and south aspects. This variability accentuates the influence of microclimatic conditions, such as sunlight exposure, temperature, and moisture levels, on regeneration success. Similarly, the elevation-dependent variations in *R. campanulatum* at Tungnath highlight the species' adaptability to higher altitudes. It shows better regeneration at 3400 m and 3450 m compared to lower elevations, which is consistent with its typical distribution in alpine environments. This pattern indicates that higher elevations provide more favorable conditions for its growth and regeneration due to reduced competition from other species and suitable soil conditions. The comprehension of these species-specific regeneration patterns is crucial for effective conservation and management strategies. The observed variations in regeneration status across species and locations indicate a need for targeted conservation efforts. For species with poor regeneration, conservation strategies should address the factors affecting their regeneration, including habitat loss, competition, and climate change impacts. Indeed, specialized management strategies that account for these variables are essential for successful conservation. This study highlights the complex interactions between climate, soil conditions and species regeneration in the Himalayan treeline, offering crucial insights for conservation and management. It emphasizes the need for targeted habitat protection and restoration efforts to support the resilience and regeneration of Himalayan treeline species amidst ongoing environmental changes.

#### 3.2.3 Soil properties of Himalayan treeline

Plant growth and development are greatly influenced by the soil characteristics. According to Liebig's law of the minimum, it is understood that the quantity of the most limiting soil nutrient, rather than the total amount of nutrients available, controls plant growth, development and seed production. The present understanding of treelines at a global scale is the result of a wide range of studies conducted on various spatial scales but soil properties in alpine treeline ecotones are insufficiently explored. Although there is much literature on treelines (mainly focusing on climate change, air temperature, change in weather patterns, and treeline shift), only a few specific soil-related studies have been conducted (e.g. Moran et al. [144]; Garkoti and Singh [197]; Gruber et al. [198,199]; Schmid et al. [200]; Liu and Luo [201]; Dawes et al. [202]; Muller et al. [203]; Rai et al. [204]; Kumar et al. [205]).

Muller et al. [203] found that soil temperature, moisture, physical properties, nutrient levels, geology, and topography influence alpine treeline vegetation globally and locally. Tree distribution (seedling, sapling and mature tree) correlates positively with nitrogen, phosphorus, potassium, and organic carbon, while negatively with sand content. Dawes et al. [202] applied a soil warming treatment during the snow-free period at the Swiss treeline and reported a +4 °C change in soil temperature between 2007 and 2012, which resulted in a significant reduction in fine roots and alterations in vegetation biomass. Liu and Luo [201] reported that soil moisture and soil temperature significantly affect the alpine treeline. Soil temperature, moisture, pH, Cu, Zn, electrical conductivity, and N levels were identified as key predictors of treeline vegetation patterns [206]. Higher soil temperatures were associated with greater species diversity across various aspects, indicating a positive relationship [76]. Stand density at the treeline varies by species due to temperature, soil, topography and microclimate [148]. Schwab et al. [186] found species-specific responses of trees in the ecotone to site conditions, with environmental factors influencing juvenile and adult trees to varying degrees. Hamid et al. [206] found that soil temperatures at the treeline edges in Kashmir Himalaya are notably higher (0.91 °C in Sinthan and 1.75 °C in Gulmarg) than the global treeline temperature average of 6.4 ± 0.7 °C. This indicates unlikely shifts in treeline positions in the Himalayan region soon, as they remain below the critical 6–7 °C isotherm. Under sufficiently moist conditions, increasing temperatures may benefit the growth of alpine trees [122].

Generally, soil nutrient availability decreases with elevation and might explain why treeline shifts and global warming are decoupled [203,205]. Nutrient levels decline with elevation, indicating soil and tree nutrient deficiency in Rolwaling's treeline ecotone. This limits tree growth, likely relying on litter recycling for plant nutrition rather than mineral soil nutrients [207]. Gupta et al. [163] studied soil properties across forest, ecotone, and meadow zones at different depths. They found higher soil moisture, pH, organic carbon, total nitrogen, and available phosphorus in meadows compared to forests might be fevering the upward shift of alpine treeline vegetation in near future. Microbial biomass varied across zones, with the ecotone showing higher values, potentially due to warming-induced shrubification in alpine ecosystems.

#### 3.2.4 Growing season of Himalayan treeline

The treeline dynamics are linked to the growing season of treeline vegetation [33,89]. The growing season of Himalayan treeline plants varies depending on the species, environmental factors and local conditions. Growth at the timberline begins in early summer as temperatures increase, with various phenological stages following one another and culminating in leaf drop as winter arrives, all influenced by changes in environmental conditions [208]. Khanduri et al. [209] investigated phenological shifts in over 650 temperate species, finding that spring events have advanced by an average of 1.9 days per decade, while autumn events have been delayed by 1.4 days per decade. Negi et al. [210] observed variations in the timing of bud break, leafing, and leaf drop among various forest species in the subtropical to temperate regions of the western Himalayas due to climate variability. Additionally, *Rhododendron arboreum* also exhibited early flowering, along with the aforementioned phenomena [112]. Mohapatra et al. [134] observed a significant increase trend in the growing season length at the rate of 12.9 days decade^−1^ in the treeline ecotone of Arunachal Pradesh. The start of the growing season has started early at the rate of −7.2 days decade^−1^ while the end of the growing season or leaf fall has been delayed at the rate of 5.7 days decade^−1^. Singh and Negi [31] investigated the impact of climate change on the periodicity of major phenophases and leaf and shoot growth dynamics over five years of *A. spectabilis*, *B. utilis*, *Q. semecarpifolia*, *R. arboreum*, and *R. campanulatum* in Tungnath treeline region of West Himalaya. The phenological event responds to an increase in atmospheric temperature by the beginning of the aforementioned stages which varies annually and these fluctuations are substantially associated with mean annual temperature. Shi and Wu [211] also reported a significant increase in the growing season at the treeline in the Sygera Mountains of eastern Tibet due to increasing temperature. Adhikari et al. [92] found that early snowmelt in a warming climate boosts species diversity in Uttarakhand Treeline ecotone, leading to advanced phenophases like vegetative, flowering, fruiting, and senescence compared to previous studies. Adhikari and Kumar [212] studied plant phenology in the western Himalayas, noting early phases like peak growth in July (75.6 %) and flowering, fruiting, and seed maturation in August (72.1 % and 23.3 %, respectively), while senescence (71 %) in September. They found shifts in phenophase timings, likely influenced by climate warming and early snowmelt, impacting microsite-level phenology. Singh et al. [213] found that treeline species' phenological and physiological activities continue despite water potential not reaching lethal levels.

## 4 Strategies for restoration and conservation of Himalayan treeline ecosystem

Climate change, particularly rising temperatures and irregular weather patterns, including unpredictable precipitation, poses a significant threat to Himalayan alpine vegetation. Developing effective conservation strategies that account for the impacts of climate change remains a considerable challenge. Beyond mitigating climate emissions, reducing greenhouse gas emissions and capturing excess atmospheric CO_2_ and GHGs are undeniably the most sustainable solutions to this crisis. If climate change continues unchecked, it may lead to elevational shifts in plant communities, disruptions in phenology, reproductive phases & reproduction, local extirpations, increased biological invasions, and the eventual degradation of the Himalayan treeline ecosystem. Monitoring the impacts of climate change and advancing research on species adaptation is essential for guiding adaptive management practices. Restoration and conservation strategies for the Himalayan treeline ecosystem should be done by protecting native species and their regeneration, maintaining soil health, restoring degraded habitats, and promoting sustainable land-use practices. Conservation efforts and their execution in alpine tree line region is crucial which should be done by raising awareness and integrating traditional knowledge through community involvement. The role of indigenous peoples and local communities is widely recognized as an essential medium for biodiversity conservation [214]. Conservation and restoration strategies that lack the support of local populations are often ineffective [215,216]. Salick et al. [69] suggested a biocultural conservation strategy that unites cultural practices and biodiversity conservation. Therefore, the active involvement of local communities must be prioritized to ensure the conservation of this unique ecosystem. Furthermore, to safeguard these fragile ecosystems, conservation strategies should include sustainable resource utilization, public awareness campaigns, enhancement of vegetation health and artificial regeneration, organized and regulated grazing practices, soil erosion control, promotion of sustainable eco-tourism, development of alternative livelihood options, biodiversity monitoring, and weed management [73,217]. A significant step towards conserving this vital region should be taken under the National Mission for Sustaining the Himalayan Ecosystem (NMSHE), a part of the National Action Plan on Climate Change, initiated by the Ministry of Science and Technology, Government of India. This mission represents a critical milestone in ensuring the preservation and sustainable development of the Himalayan treeline ecosystem.

## 5 Conclusion:

The alpine tree line in the Himalayas represents a critical ecological habitat that harbours a diverse flora and at the same time serves as a sensitive indicator of the effects of climate change. The effects of climate change on the Himalayan alpine tree line are complex and are influenced by the factors such as temperature rise, precipitation patterns and topography, with species responses and widely varying tree line dynamics. Rising temperatures and changing precipitation patterns have led to upward shifts in the positions of the treeline in different regions of the Himalayas. Species such as *Rhododendron campanulatum* and *Abies spectabilis* are particularly sensitive to these climatic changes, with *Rhododendron campanulatum* having a strong potential for upward expansion due to its competitive advantage in the krummholz zones, possibly hindering the migration of other tree species. In addition, changes in snowmelt patterns affect plant communities, favouring warm-adapted species and possibly leading to the loss of cold-adapted species. The regeneration dynamics of tree species within the Himalayan alpine treeline are complex and influenced by several factors, including climate change, soil characteristics and elevation. The report highlights that some species, such as *Rhododendron campanulatum*, show strong regeneration patterns at higher elevations, while others such as *Betula utilis* and *Quercus semecarpifolia* struggle to regenerate effectively at different sites due to unfavourable conditions. Furthermore, the effects of climate change are evident; changes in growing seasons and higher temperatures contribute to shifts in the treeline dynamics. Understanding the complex interactions between climate, soil properties and species regeneration is crucial for developing effective management plans to ensure the resilience and sustainability of Himalayan treeline ecosystems in the face of ongoing environmental changes. The review underscores the need for targeted conservation strategies that address these specific challenges, including habitat protection and restoration efforts tailored to species-specific requirements. Understanding the intricate interactions between treeline vegetation, climate, soil properties and species regeneration is crucial for developing effective management plans to ensure the resilience and sustainability of Himalayan treeline ecosystems in the face of ongoing environmental changes.

## CRediT authorship contribution statement

**Sandeep Kumar:** Writing – original draft, Formal analysis, Data curation, Conceptualization. **Vinod Prasad Khanduri:** Writing – review & editing, Validation, Supervision, Conceptualization.

## Submission declaration and verification –

The study is not under consideration for publication elsewhere and its publication is approved by all authors and tacitly or explicitly by the responsible authorities where the work was carried out, and that, if accepted, it will not be published elsewhere in the same form, in English or in any other language, including electronically without the written consent of the copyright holder.

## Data statement –

The data will be made available on request from the corresponding author.

## Funding sources –

This study is not supported financially by any funding agency.

## Declaration of competing interest

The authors declare that they have no known competing financial interests or personal relationships that could have appeared to influence the work reported in this paper.
